# Developing Body-Components-Based Theranostic Nanoparticles for Targeting Ovarian Cancer

**DOI:** 10.3390/pharmaceutics11050216

**Published:** 2019-05-05

**Authors:** Ravit Edelman, Yehuda G. Assaraf, Anton Slavkin, Tamar Dolev, Tal Shahar, Yoav D. Livney

**Affiliations:** 1 The Lab of Biopolymers for Food and Health, Department of Biotechnology and Food Engineering, Technion-Israel Institute of Technology, Haifa 3200000, Israel; ravited@technion.ac.il (R.E.); Anton.Slavkin@Strauss-Group.com (A.S.); tdolev@gmail.com (T.D.); talshahar77@gmail.com (T.S.); 2 The Fred Wyszkowski Cancer Research Laboratory, Department of Biology, Technion-Israel Institute of Technology, Haifa 3200000, Israel

**Keywords:** hyaluronic acid, serum albumin, Maillard conjugates, theranostic nanoparticles, CD44-targeted chemotherapy, ovarian cancer

## Abstract

Ovarian cancer mortality is the highest among gynecologic malignancies. Hence, the major challenges are early diagnosis and efficient targeted therapy. Herein, we devised model theranostic nanoparticles (NPs) for combined diagnostics and delivery of chemotherapeutics, targeted to ovarian cancer cells. These NPs were made of natural biocompatible and biodegradable body components: hyaluronic acid (HA) and serum albumin (SA). The hydrophilic HA served as the targeting ligand for cancer cells overexpressing CD44, the HA receptor. SA, the natural carrier of various ligands through the blood, served as the hydrophobic block of the self-assembling block copolymeric Maillard-conjugates. We show the successful construction of fluorescently-labeled SA-HA conjugate-based theranostic NPs, their loading with paclitaxel (PTX) (association constant (8.6 ± 0.8) × 10^3^ M^−1^, maximal loading capacity of 4:1 PTX:BSA, and 96% encapsulation efficiency), selective internalization and cytotoxicity to CD44-overexpressing ovarian cancer cells (IC_50_: 26.4 ± 2.3 nM, compared to 115.0 ± 17.4 of free PTX, and to 58.6 ± 19.7 nM for CD44-lacking cognate ovarian cancer cells). Fluorescein isothiocyanate (FITC) was used for in vitro imaging, whereas long wavelength fluorophores or other suitable tracers would be used for future in vivo diagnostic imaging. Collectively, our findings demonstrate that fluorescent HA-SA NPs harboring a cytotoxic drug cargo can specifically target, label CD44-expressing ovarian cancer cells and efficiently eradicate them.

## 1. Introduction

Cancer is a global disease, which causes 8.2 million human deaths each year. Five percent of all cancer deaths among women are attributable to ovarian cancer [1]. One of the main challenges in the combat against cancer is the precise diagnostic localization and treatment of the tumor and its distant metastases. Conventional chemotherapy inflicts severe untoward effects and displays unsatisfactory efficacy due to the frequent emergence of multidrug resistance (MDR), often due to multidrug efflux transporters, like P-glycoprotein (P-gp) [2,3,4] and novel MDR modalities including lysosomal drug sequestration of an array of chemotherapeutics [5,6,7,8]. Anti-microtubule agents of the class of Taxanes, including paclitaxel (PTX), docetaxel and cabazitaxel, are effective hydrophobic chemotherapeutic drugs that are widely used in the treatment of a spectrum of carcinomas including ovarian, breast, lung, stomach, prostate and head and neck cancers [9]. Due to its poor water-solubility, PTX solubilization is required [8]. Moreover, given the lack of PTX selectivity, relatively high drug doses are administered, causing severe untoward toxicity. Moreover, various mechanisms of chemoresistance have been documented to these anti-microtubule agents. To overcome these impediments, a major goal of drug delivery systems (DDS) is to carry the drug selectively to the target tumor, achieve intracellular drug internalization and tumor cell eradication. Selective delivery of nanovehicles to malignant tumors is highly desirable to minimize side effects and maximize drug selectivity and efficacy [2,3,10]. Passive targeting into tumors may be achieved by long-circulating nanoparticles (NPs) [5,11], entering the tumor through its leaky vasculature, and remaining there due to its poor lymphatic drainage, based on the enhanced permeability and retention (EPR) effect [2,12].

In recent years, a number of clinical trials have been conducted, examining a number of treatments for ovarian cancer. Recently, the addition of bevacizumab, a VEGF-targeted antibody, which blocks angiogenesis, to the common combination treatment of carboplatin and paclitaxel was examined in a phase III clinical trial. The addition of bevacizumab significantly improved the median progression-free survival to 19.4 months compared to 17.3 months in the standard therapy [11]. Another phase II clinical trial that was conducted recently investigated the effect of a combination of Olaparib (PARP inhibitor) with the standard treatment for patients with platinum-sensitive, recurrent, high-grade serous ovarian cancer [13]. Addition of Olaparib to PTX and carboplatin improved the progression-free survival [13].

Polymeric micelles are typically formed of block copolymers, which self-assemble in aqueous solutions. Active-targeting molecules, including certain vitamins, hormones, carbohydrates, peptides, antibodies, or nucleic acid aptamers, have been conjugated to nano-carrier surface for selective delivery to cancer cells [3,14]. Internalization of the NPs into cancer cells via receptor-mediated endocytosis results in both enhanced cellular uptake and anti-tumor activity [15]. Various normal tissues express CD44 at low levels and bind hyaluronic acid (HA) with low affinity. Many human carcinomas with high metastatic capacity including breast, ovarian, colon, lung and stomach cancers, are characterized by upregulation of CD44 expression on the cell surface [16,17,18,19]. HA is a physiological body constituent, and it is biocompatible, biodegradable and non-immunogenic [20]. Many efforts have been invested for the development of HA-based micelles, due to the efficient binding of HA to its receptor, CD44, and the release of the drug cargo after internalization into the tumor [15,21,22,23,24]. For example, Bae et al., conjugated HA to EGCG for encapsulation of cisplatin [21]. Whereas, Jung Cho et al., created a gel composed of adipic dihydrazide modified HA with oxidized HA for sustained release of cisplatinum [25].

In the fluorescently labeled SA-HA conjugate, HA serves as both the active targeting component and the hydrophilic external domain in the amphiphilic nanocarrier. By using HA-based targeted nanovehicles, the selectivity towards cancer cells is markedly enhanced, suggesting that overall drug dosage could be reduced [20,26]. Serum albumin (SA) is the major natural nanovehicle of various compounds in mammalian blood. Human serum albumin (HSA) has a molecular weight of 66.5 kDa; SA is a physiological carrier for proteins and lipids present in blood plasma; it was thus selected as the appropriate protein for binding hydrophobic drugs [27,28]. SA has been applied in various DDS, e.g., Abraxane^®^, which is a paclitaxel (PTX), stabilized within HSA NPs [29]. HSA NPs are biocompatible, biodegradable and non-immunogenic [29]. Herein we used bovine serum albumin (BSA) as a model, although in the future, towards clinical evaluation stages, we plan to use HSA.

Multifunctional NPs integrate various functionalities inside the core and/or on the surface of NPs to synergistically achieve maximal anti-tumor activity [2,30]. The surface of NPs can be functionalized with ligands that can specifically target receptors on tumor cell surface for selective targeted drug delivery [30,31], such as small molecule ligands (e.g., folic acid [32,33]), or biopolymers such as aptamer sequences [30], antibodies [34,35] and HA [36]. Theranostic NPs may simultaneously serve in cancer diagnostics and therapeutics. A variety of materials including synthetic polymers [37,38,39,40], graphene [41,42], proteins [35,43], polysaccharides [44], lipids [37,45], quantum dots [46,47] and metallic NPs [48,49,50], organized in different structures (e.g., micelles [51,52], vesicles [53,54], dendrimers [55], nanorods [48], nanotubes [56,57,58], nanosheets [38] and more) have been used to develop theranostic NPs. The vast majority of nanomedical vehicles proposed in the literature is based on synthetic or otherwise exogenous materials, and it would be highly advantageous to design and construct nanovehicles from natural body components, for better biocompatibility, non-immunogenicity and biodegradability. Several theranostic particles composed of HA or albumin were developed and evaluated [23,24,59]. However, such HA-SA conjugates [36] have not been proposed by other groups, and the fluorophore-HA-SA conjugates are being introduced here for the first time. Although there are several promising treatments for ovarian cancer being developed every year, the 5 year survival remains lower than 42% [60].

Herein we propose a new theranostic combination of three crucial components: (a) a targeting moiety (HA), (b) a cytotoxic drug cargo (PTX), and (c) a diagnostic tracer (FITC) within the same biocompatible nanovehicle. FITC was used for in vitro imaging, whereas long wavelength fluorophores or other in vivo suitable tracers would be used for future diagnostic imaging. The aim of the current study was to form prototype theranostic tracer-HA-SA conjugate-based NPs, characterize their self-assembly, drug loading and selective cytotoxicity to human ovarian cancer cells overexpressing CD44. These NPs should allow the targeted delivery of lower doses of cytotoxic drugs, hence diminishing untoward toxicity to healthy tissues. By entering the cell via endocytosis, the drug cargo may overcome impaired drug influx and evade enhanced drug efflux via MDR extrusion pumps, hence should be effective in overcoming MDR [2,3,61,62,63]. Additionally, once labeled with tracers for in vivo imaging, they would facilitate imaging and localization of the tumor and its distant metastases, to enable accurate and efficacious complementary treatments such as radiotherapy, photodynamic therapy and surgery [2].

Herein, we have successfully conjugated the fluorescent model tracer FITC to HA and the latter to BSA and formed self-assembling, actively targeted biopolymeric nanocarriers. We have loaded them with PTX and demonstrated their selective uptake by CD44-overexpressing human ovarian cancer cells followed by eradication of these malignant cells.

## 2. Materials and Methods

### 2.1. Materials

Sodium hyaluronan (6.4 kDa) was purchased from Lifecore Biomedical, LLC (Chaska, MN, USA). BSA, PTX, FITC and Hoechst 33342 were acquired from Sigma-Aldrich Ltd. (Rehovot, Israel). DMSO was from Loba Chemie (Mumbai, India). Human ovarian adenocarcinoma cell lines SKOV3 and A2780 were generously provided by Ilana Chefetz-Menaker, the Lab of Gil Mor, Yale University, New Haven, CT, USA. McCoy’s 5A, RPMI-1640, 10% fetal bovine serum media and XTT colorimetric cell proliferation kit were purchased from Biological Industries, Beit-Haemek, Israel. 1,6-diphenyl-1,3,5-hexatriene (DPH) was kindly provided by Boaz Mizrahi, Biotechnology and Food Engineering Department, Technion IIT.

### 2.2. Methods

#### 2.2.1. Preparation of Fluorescein Isothiocyanate-Hyaluronic Acid-Bovine Serum Albumin (FITC-HA-BSA) Conjugates

Adipic dihydrazide-fluorescein isothiocyanate (ADH-FITC) conjugate was synthesized based on the protocol of El-Dakdouki et al., [64]. The synthesis of the FITC-ADH-HA conjugate was examined at a FITC: HA molar ratio of 1:5. HA (6.4 kDa, 100 mg) and was dissolved in high-performance liquid chromatography (HPLC)-grade water (0.26 mL), and FITC-ADH (1.5 mg) was added. The pH of the solution was adjusted to 4.5–4.8 by the dropwise addition of a 10 mM aqueous HCl solution. 1-Ethyl-3-(3-dimethylaminopropyl) carbodiimide (EDCI) (1.5 mg) was added, and the solution was stirred at room temperature for 9 h during which the pH was constantly adjusted to 4.5–4.8. The reaction mixture was then brought to pH 7.0 by adding a 10 mM NaOH solution. To minimize the amount of free FITC in the reaction mixture, the sample was dialyzed (MWCO 2 kDa) against deionized water overnight at 4 °C. After FITC conjugation of HA, FITC-HA-BSA conjugates were prepared as previously described [36].

#### 2.2.2. Determination of the Critical Micellization Concentration (CMC)

The critical micellization concentration (CMC) of FITC-HA-BSA conjugates was determined using a hydrophobic dye solubilization method using DPH as a probe [65]. Samples of FITC-HA-BSA conjugates were prepared at different concentrations (0.01–2.9 mg/mL) at a constant concentration of DPH (1%). The absorbance at 378 nm relative to that at 400 nm was plotted against the concentration of FITC-HA-BSA conjugates. The intersection point of the two extrapolated lines was defined as the CMC of the NPs.

#### 2.2.3. Preparation of FITC-HA-BSA–Paclitaxel Nanoparticles (PTX NPs)

FITC-HA-BSA conjugates were dissolved in phosphate buffered saline (PBS, pH 7) and PTX was dissolved in DMSO. Then, encapsulation of PTX within a FITC-HA-BSA conjugate solution was achieved by dropwise addition of the PTX-DMSO stock into the conjugate solution, while continuously stirring. Samples were shaken for one additional h for equilibration.

#### 2.2.4. Size Determination

The diameter of FITC-HA-BSA conjugates (40 µM), PTX (80 µM) and the encapsulated PTX (molar ratio of 2:1 PTX:FITC-HA-BSA) in PBS (pH 7.4) were determined using a dynamic light scattering (DLS) analyzer (NicompTM 380, PSS Inc., Santa Barbara, CA, USA).

#### 2.2.5. Zeta Potential

The electrophoretic mobilities of unencapsulated PTX, PTX encapsulated within FITC-HA-BSA conjugates and FITC-HA-BSA conjugates alone were determined by using a Zetasizer Nano instrument (Malvern Instruments Ltd., Worcestershire, UK). The zeta potential calculation was based on the Smoluchowski model.

#### 2.2.6. Light Microscopy

Light microscopy images of unencapsulated 80 µM PTX in 20 mM phosphate buffered saline at pH 7.4, containing 0.8% DMSO, were compared to those obtained at the same concentration of PTX, with FITC-HA-BSA conjugates (molar ratios of 12:1 to 2:1 of PTX: FITC-HA-BSA conjugates). The maximal loading capacity (LC) of FITC-HA-BSA was determined using light microscopy images. Those were taken at X40 magnification using an Olympus X51 light microscope, operated in a bright-field optical mode.

#### 2.2.7. Cryogenic-Transmission Electron Microscopy (Cryo-TEM) Imaging

Cryogenic-transmission electron microscopy (Cryo-TEM) imaging was performed using two samples: 1. FITC-HA-BSA conjugates (40 µM); 2. Encapsulated PTX (80 µM) in FITC-HA-BSA conjugates (PTX:FITC-HA-BSA NPs molar ratio of 2:1). Samples were prepared as previously described [36]. Briefly, the specimens were prepared on carbon film (10–500 nm film), frozen in liquid ethane, and stored under liquid nitrogen. The specimens were observed in a Philips CM120 electron microscope) FEI-Thermo-scientific, Hillsboro, OR, USA).

#### 2.2.8. Encapsulation Efficiency (EE)

To determine the encapsulation efficiency (EE) of PTX loaded within FITC-HA-BSA NPs, a sample of 1 mg/mL of NPs, on a protein basis, was prepared to contain PTX at the maximal LC. The samples were centrifuged at 8000× *g* for 20 min. The pellet was collected, and the concentration of PTX was determined using reversed phase HPLC (RP-HPLC). Samples were analyzed using a 4.6 × 250 mm C18 RP-HPLC column. The mobile phase consisted of acetonitrile and ammonium acetate buffer solution (10 mM, pH 5.0) (50:45, *v*/*v*).

Analysis of EE was performed using Equation (1):

(1) $$\mathrm{Encapsulation}\;\mathrm{Efficiency}\;\left(\mathrm{EE}\right)\;\left(\%\right)=\frac{\mathrm{PTX}\_\mathrm{Encapsulated}}{\mathrm{PTX}\_\mathrm{total}}\cdot 100$$

#### 2.2.9. Binding Studies by Spectrophotometry

FITC-HA-BSA conjugates (40 µM) and (80 µM) PTX-loaded FITC-HA-BSA NPs (40 µM) were prepared as described above. The absorbance spectra of these samples, and of the dispersion of PTX alone (stock solution added to buffer only), were measured. The summation of the absorbance of PTX and of FITC-HA-BSA conjugates was calculated and compared to the absorbance of (80 µM) PTX-loaded FITC-HA-BSA NPs (40 µM). The spectroscopic measurements were performed using Evolution 201, UV-Visible spectrophotometer (Thermo Scientific, Bargal, Shoham, Israel).

#### 2.2.10. Binding Studies by Spectrofluorometry

Quenching of BSA tryptophan was used to study the binding of PTX to BSA. Encapsulation of PTX at increasing concentrations (0–80 µM) at a constant BSA concentration (40 µM) was performed as described above. BSA fluorescence was determined using the Fluorolog 3-22 spectrofluoremeter (Horiba, Jobin Yvon, Longjumeau, France) at a right-angle mode.

The analysis for determination of the binding constant (Ka) of PTX to BSA-HA was performed using a Langmuir-based model, assuming one affinity-class of binding sites, as described by Equation (2) [66]:

(2) $$F-F_{0}=L_{0}\left(\frac{F_{0}-F_{\infty }}{\frac{1}{K_{a}}+L_{0}}\right)$$

where *F*, *F*_0_ are BSA measured fluorescence intensity in the presence and absence of PTX, respectively. *F*_∞_ is the fluorescence when the protein is saturated. *L*_0_ is the total concentration of PTX.

#### 2.2.11. In Vitro PTX Release

FITC-HA-BSA was dissolved in PBS (pH 7.4) at a concentration of 100µM. PTX was added to the solution at a 2:1 molar ratio while continuously stirring. FITC-HA-BSA conjugate-PTX solution was transferred to GeBaFlex-tubes (Dialysis kit of 3.5 kDa MWCO) and inserted into falcons with release medium containing 30 mL PBS, with or without 0.1% (*v*/*v*) Tween-80. At time intervals, during 80 h, 2.7 mL aliquots were taken and replaced with fresh Tween-80-PBS or a plain PBS solution, respectively. PTX concentration was determined as described above using RP-HPLC.

#### 2.2.12. Stability of FITC-HA-BSA Conjugates

PTX-loaded FITC-HA-BSA conjugate NPs solution (in PBS) (PTX:FITC-HA-BSA conjugate molar ratio of 2:1) were challenged in two ways: dissolution in 50% FBS (fetal bovine serum) and dissolution in a solution of sodium dodecyl sulfate (SDS) (43.2 g/L, 10:1 weight ratio SDS: FITC-HA-BSA conjugates). The stability of the PTX-loaded NPs under these harsh conditions was studied by measuring the relative scattered light intensity (SLI_t_/SLI_0_) and the mean diameter [15,67]. The relative scattered light intensity was calculated as the intensity in time t (SLI_t_) compared to the initial intensity (SLI_0_). Measurements were performed during 3 h using DLS.

#### 2.2.13. Tissue Culture

Human ovarian adenocarcinoma cell lines SKOV3 and A2780 were grown in McCoy’s and RPMI-1640 media, respectively, and supplemented with 10% fetal bovine serum and 1 mM L-glutamine. These tumor cells were grown under a humidified atmosphere of 5% CO_2_ at 37 °C.

#### 2.2.14. Selective Targeting

The selective targeting of FITC-HA-BSA conjugates to human ovarian adenocarcinoma cell lines SKOV3 and A2780 was examined using a Zeiss inverted Cell-Observer microscope as previously described [36]. Briefly, two days before the experiment, 2 × 10^4^ cells/2 mL were seeded and cultured in growth medium. The relevant samples were added 2 h prior to cell imaging. In addition, LysoTracker^®^ Red was added after 1 h for lysosome labeling, whereas Hoechst 33342 was added immediately before imaging for nuclear DNA staining. Three samples were studied on each tumor cell line: (1) FITC-HA-BSA NPs (20 µM); (2) (40 µM) PTX loaded FITC-HA-BSA NPs (20 µM); (3) (40 µM) PTX loaded FITC-HA-BSA NPs (20 µM) with excess of free HA (50 mg/mL compared to 0.5 mg/mL conjugated HA, at a pulse exposure of the cells to free HA for 1 h). This 100-fold higher concentration than the HA-conjugated NPs did not display any deleterious effect on the cells, and cells were viable during the entire experiment. This concentration was selected while also considering that even a 100 mg/mL HA had been reported to be safe for a human cell culture, and was even able to promote physiological differentiation to cartilage cells [68]. Internalization of FITC-HA-BSA NPs into lysosomes, via CD44 receptor-mediated endocytosis was demonstrated by the yellow fluorescence resulting from co-localization of LysoTracker^®^ Red and the green FITC fluorescent dyes.

#### 2.2.15. Cytotoxicity Assays

Cytotoxic activity was determined using an XTT-based colorimetric cell proliferation kit. Cells were seeded at 1 × 10^4^ cells/well in 96-well plates containing 100 µl growth medium/well. Following overnight incubation, cells were exposed to either: PTX or PTX encapsulated within FITC-HA-BSA conjugate NPs. Excess of free HA (100:1 molar ratio of free HA to conjugated HA NPs). Incubation time for PTX (10 µL/well) was 2 h. Cellular viability was determined using a colorimetric XTT cell proliferation assay. After dissolving the formed formazan dye, absorbance was measured by a microplate reader. Cytotoxicity data were analyzed with OriginPro 8.6, using a non-linear curve fitting. IC_50_ is the drug concentration that inhibits cell growth by 50%. Results shown are means ± standard error (SE) obtained from three independent experiments, each performed in triplicates.

#### 2.2.16. Statistical Analysis

Each experiment was performed in triplicates, and means as well as standard error (SE) were calculated. The statistical analysis of significance of the difference between each two groups in the cytotoxicity assay was evaluated using a two-sample *t*-test, assuming equal variances. Analysis was performed using Microsoft Excel 2013™.

## 3. Results

### 3.1. Characterization of FITC-HA-BSA Conjugates

#### Conjugation of FITC-HA to BSA

**Figure 1:**
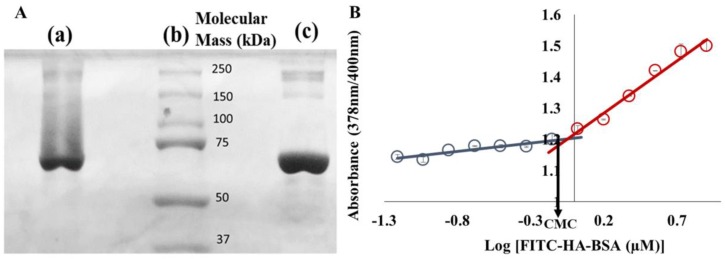
(**A**) Sodium dodecyl sulfate polyacrylamide gel electrophoresis (SDS-PAGE) of: (**a**) a solution of fluorescein isothiocyanate-hyaluronic acid-bovine serum albumin (FITC-HA-BSA) conjugates, (**b**) standard MW markers, and (**c**) BSA; (**B**) Relative 1,6-diphenyl-1,3,5-hexatriene (DPH) absorbance at 378 nm and 400 nm vs. increasing concentrations of FITC-HA-BSA. The final concentration of DPH was 1%. The critical micellization concentration (CMC) (1.1 µM) was determined as the intersection point of the two extrapolated lines.

FITC-HA-BSA conjugates were prepared; Figure 1A displays sodium dodecyl sulfate polyacrylamide gel electrophoresis (SDS-PAGE) results that determined the molecular weight of FITC-HA-BSA conjugates compared to BSA alone, at the same BSA concentration. Figure 1B describes the ratio of DPH absorbance values from 378 nm to 400 nm, at different FITC-HA-BSA conjugate concentrations.

Figure 1A describes an SDS-PAGE analysis to assess the molecular mass of FITC-HA-BSA conjugates compared to BSA alone. The major band of FITC-HA-BSA conjugates is similar to the principal band of BSA alone (~67 kDa—Figure 1A, lane c). During the Maillard reaction, BSA was covalently attached to FITC-HA, thus the molecular mass increased (the average molecular mass observed was ~85 kDa, Figure 1A, lane a). The CMC of FITC-HA-BSA was evaluated using the fluorescent compound 1,6-diphenyl-1,3,5-hexatriene (DPH) as a probe for hydrophobic domains. DPH is a hydrophobic molecule, whose absorbance is mostly affected by the hydrophobicity of the nanoenvironment it is dissolved or entrapped in [69]. Figure 1B shows that the 378/400 nm absorbance ratio of DPH at low FITC-HA-BSA concentrations was hardly changed. Increasing concentrations above 1.1 µM FITC-HA-BSA caused a steeper increase in the absorbance ratio. The CMC of FITC-HA-BSA conjugates was thus determined as 1.1 µM, which is derived as the concentration at the intersection point of the two extrapolated line fits.

### 3.2. Encapsulation of PTX within FITC-HA-BSA Conjugates

Analysis of the encapsulation of PTX within FITC-HA-BSA conjugates was performed by evaluating the association constant, the particle size distribution, and the drug LC and EE.

#### 3.2.1. Association Constant Determination

**Figure 2:**
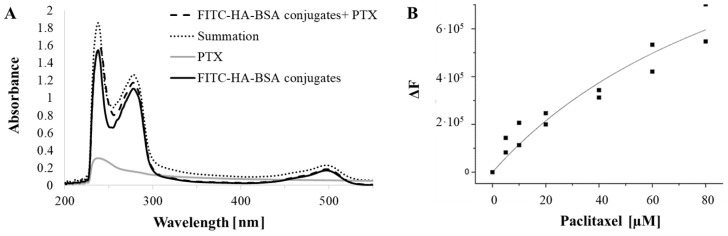
(**A**) Absorbance of FITC-HA-BSA conjugates (40 µM; black solid line); of (80 µM) PTX encapsulated in the conjugate NPs (dashed line), (PTX: FITC-HA-BSA conjugate molar ratio of 2:1), of pure PTX (80 µM, gray solid line) and the absorbance summation of FITC-HA-BSA conjugates and PTX (dotted line); (**B**) Fluorescence quenching of HA-BSA by PTX as a function of PTX concentration. The excitation and emission were at 290 and 350 nm, respectively. The curve is a fit to a Langmuir-based model as described in Equation (2).

The binding of the FITC-HA-BSA conjugates to PTX was studied by spectrophotometry. The absorbance spectra of FITC-HA-BSA conjugates, PTX and PTX encapsulated within FITC-HA-BSA are described in Figure 2A. Quenching of the intrinsic fluorescence of BSA tryptophans was used to quantify the association constant of PTX to BSA. Trp 135 and Trp 214 are the two tryptophan residues of BSA; Trp 135 is more exposed to the hydrophilic nanoenvironment, whereas Trp 214 (also present in HSA) is engulfed in a hydrophobic loop of BSA. PTX is a hydrophobic molecule, thus is attracted to the hydrophobic subdomain IIA of BSA, where Trp 214 is positioned. Changes in tryptophan fluorescence spectra of HA-BSA in the presence of PTX are presented in Figure 2B.

Figure 2A shows that the absorbance summation of FITC-HA-BSA conjugates and PTX was higher than the absorbance of PTX encapsulated in the conjugate NPs, indicating a binding interaction between PTX and FITC-HA-BSA conjugates. The difference between the absorbance at the wavelengths 237 nm (the maximum absorbance of PTX) and 497 nm (the maximum absorbance of FITC) was significant (*p*-value < 0.05). It is evident from Figure 2B that the intrinsic fluorescence intensity of HA-BSA is decreasing with increasing PTX concentrations. PTX interacts with hydrophobic protein subdomains containing tryptophan residues.

The intrinsic tryptophan fluorescence of BSA is quenched by PTX, which reflects an interaction between PTX and BSA. The association constant (Ka) of HA-BSA NPs calculated from the non-linear curve fit of the Langmuir model was found to be (8.6 ± 0.8) × 10^3^ M^−1^.

#### 3.2.2. Particle Size Determination

**Figure 3:**
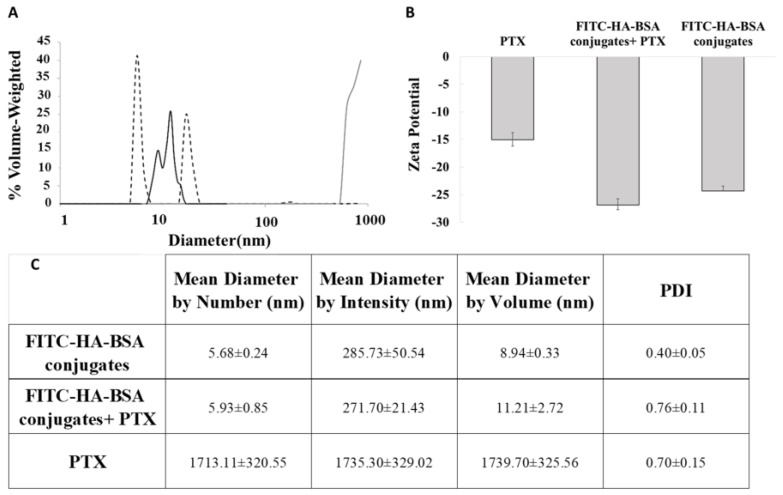
(**A**) Particle size distributions of FITC-HA-BSA conjugates and their self-assemblies (40 µM; black solid line); of (80 µM) PTX-loaded conjugate NPs (dashed line), (PTX: FITC-HA-BSA conjugate molar ratio of 2:1), and of pure PTX (80 µM; gray solid line). (**B**) Zeta potential of FITC-HA-BSA conjugates (40 µM) and of (80 µM) PTX-loaded conjugate NPs. (**C**) PDI and mean diameter by number, intensity and volume of the three samples.

Particle size distribution analysis of the three samples: FITC-HA-BSA conjugates, PTX and encapsulated PTX, is displayed in Figure 3.

**Figure 4:**
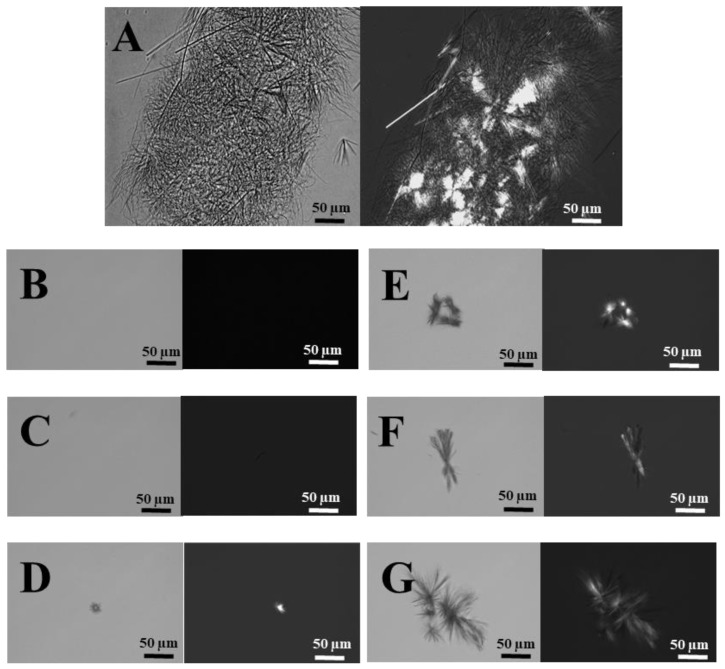
Bright field (**left**) and polarized light (**right**) microscopy images (scale bars = 50 µm) of (**A**) non-encapsulated PTX (80 μM); (**B**) 2:1, (**C**) 4:1, (**D**) 6:1, (**E**) 8:1, (**F**) 10:1, (**G**) 12:1 molar ratio of PTX to FITC-HA-BSA conjugate NPs.

The diameters of FITC-HA-BSA (Figure 3A, trimodal solid line) conjugates were 5–8 nm (left-most peak), and their self-assemblies were ~10−20 nm (second and third peaks). The diameter of PTX as determined by the DLS was ~1 µm, due to the measurement limit. The real diameter of pure PTX particles was above 50 µm as shown in the light microscopy image (Figure 4A). However, FITC-HA-BSA conjugate NPs in the presence of PTX exhibited two nanometric populations: one of ~4 nm and another of ~20 nm (Fig 3A, dashed line). Hence, the diameters of FITC-HA-BSA conjugate NPs were similar, on average, in the presence and absence of PTX. Moreover, the absolute zeta potential value of PTX alone was lower than that of FITC-HA-BSA conjugates (Figure 3B). Higher absolute value of zeta potential indicates better colloidal stability of the sample. The loading with PTX did not change the zeta potential of the conjugate NPs, indicating that the encapsulation does not affect the stability of the NPs. However, the encapsulation increased the absolute value of the zeta potential of PTX, hence it dramatically improved its colloidal stability.

Figure 4 displays the light microscopy images of: (A) Non-encapsulated PTX in PBS containing 0.8% DMSO; (B–F) PTX entrapped within BSA-HA conjugates at 2:1 to 12:1 molar ratios, containing 0.8% DMSO.

Figure 4A shows that PTX formed micrometer-sized aggregates of needle-like crystals visualized by light microscopy. FITC-HA-BSA conjugates and their assemblies are smaller than 15 nm (Figure 3A), thus are not visible in light microscopy. Encapsulation of PTX within FITC-HA-BSA conjugates suppressed crystal growth at molar ratios above 4:1 (Figure 4D–G) and apparently prevented the formation of visible microcrystals up to 4:1 molar ratio (Figure 4B,C).

**Figure 5:**
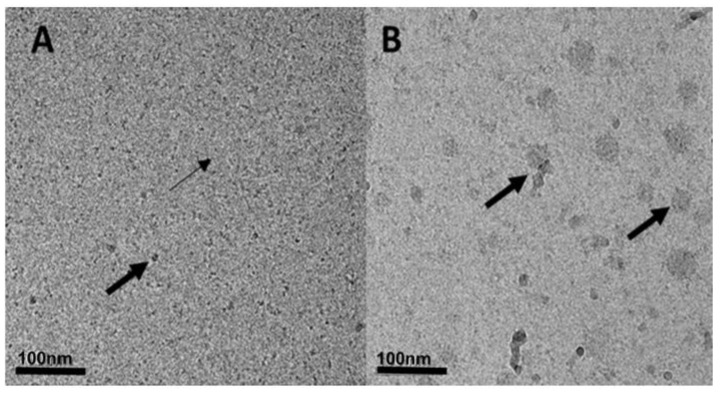
Cryogenic-transmission electron microscopy (Cryo-TEM) images of (**A**) FITC-HA-BSA NPs (40 μM). Small arrows indicate the FITC-HA-BSA NPs and large arrows indicate self-assembled aggregates of FITC-HA-BSA NPs. (**B**) Encapsulated PTX with a molar ratio of 2:1 PTX: FITC-HA-BSA conjugates; arrows indicate PTX aggregates and nanocrystals, to which FITC-HA-BSA NPs seem to be adsorbed.

Figure 5 displays Cryo-TEM images of FITC-HA-BSA conjugates and FITC-HA-BSA conjugates encapsulating PTX at a molar ratio of 2:1 PTX to FITC-HA-BSA.

**Figure 6:**
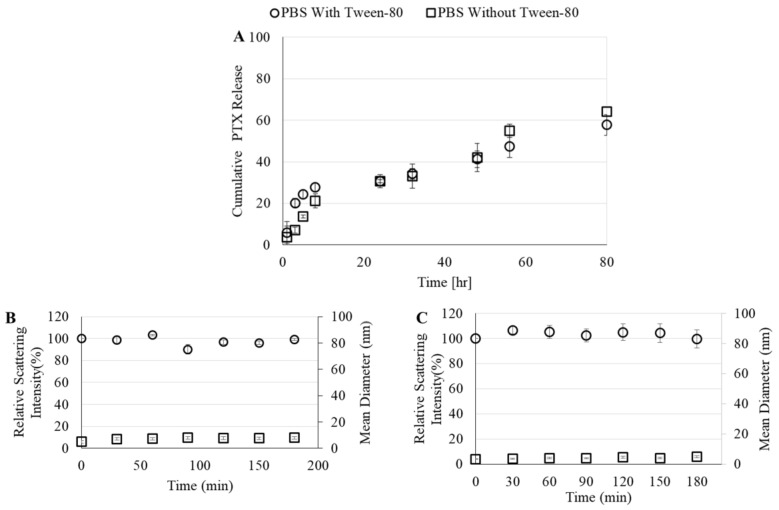
(**A**) Cumulative drug release from FITC-HA-BSA conjugates to phosphate buffered saline (PBS) with or without 0.1% Tween 80. Relative scattering intensity and mean diameter of FITC-HA-BSA conjugates with PTX in the presence of (**B**) 50% fetal bovine serum (FBS), or (**C**) sodium dodecyl sulfate (SDS) (43.2 gm/mL).

Cryo-TEM images indicate that the FITC-HA-BSA conjugates were mostly around ~5–7 nm (based on the DLS, Figure 3A, left-most peak); this can be seen by the small arrows in Figure 6A. Moreover, the larger NPs observed (10–20 nm, second and third peaks in Figure 3A, solid line, and Figure 5A, large arrows) may indicate that FITC-HA-BSA conjugates are self-assembled. In Figure 5B, the self-assembled FITC-HA-BSA NPs have entrapped PTX within their hydrophobic core, or were adsorbed onto surfaces of excess PTX aggregates and crystals. Addition of PTX to a FITC-HA-BSA conjugate solution caused an increase in the average particle size. The large arrows indicate small needle-like nanocrystals of PTX at a molar ratio of 2:1 PTX: FITC-HA-BSA (Figure 5B). FITC-HA-BSA NPs prevent extensive aggregation of PTX in aqueous solutions creating colloidally stable nanometric particles.

#### 3.2.3. Drug-Loading Capacity (LC) and Encapsulation Efficiency (EE)

Drug LC and EE are important indices for drug delivery systems as one would like to efficiently obtain a substantial drug cargo in the loaded NP. The maximal LC was evaluated as the highest ratio at which no crystals were visible by light microscopy. This was found to be at approximately a molar ratio of 4:1 PTX: FITC-HA-BSA (on a protein basis); this translates into a mass ratio of 33 mg PTX per gm BSA. The EE was quantified by centrifugally sedimenting the unentrapped excess PTX at the point of maximal LC, and was found to be 96%.

#### 3.2.4. PTX Release

Cumulative PTX release from FITC-HA-BSA NPs to two different release media (with and without Tween 80) is demonstrated in Figure 6.

The maximal release of PTX from FITC-HA-BSA NPs was 60%, regardless of the presence of Tween 80 in the drug-release medium. An initial burst release occurred in the first 10 h and caused 20% release of the initial amount of PTX (Figure 6A). Then, the drug-release rate subsided, and after 24 h (a typical time scale required for the EPR effect [70]) more than 70% of the drug remained encapsulated. The relative light scattering intensity of PTX-loaded FITC-HA-BSA conjugate NPs almost did not change in the presence of FBS or even SDS. Even after 3 h, the relative intensity was almost 100%. In addition, the mean diameter of the NPs in the presence of FBS slightly increased (6.4 nm to 8 nm) due to interaction of FITC-HA-BSA conjugates with the albumin present in the serum (Figure 6B). In the presence of SDS, the mean diameter was lower than that of FITC-HA-BSA conjugates alone due to addition of a high concentration SDS (43.2 gm/L) which creates smaller micellar particles (Figure 6C).

#### 3.2.5. Selective Targeting to CD44-Overexpressing Tumor Cells

**Figure 7:**
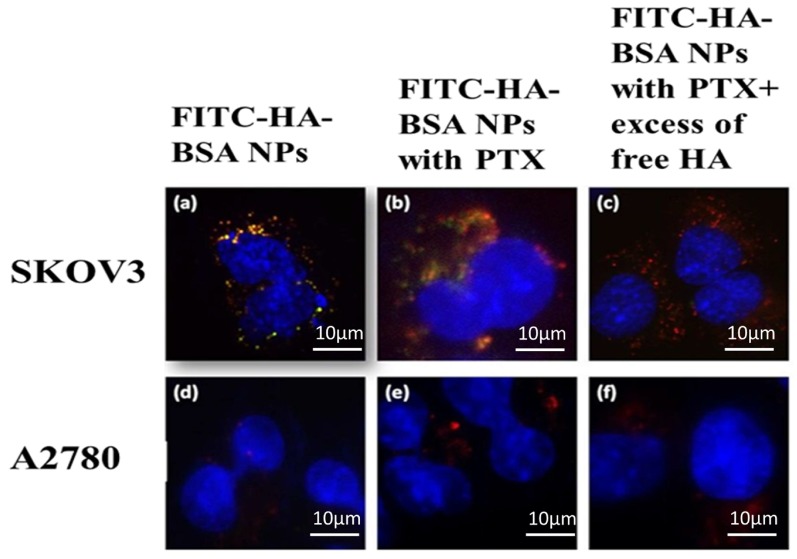
SKOV3 (CD44+) and A2780 cells (CD44−) were incubated with 3 different NPs: (**a**,**d**) FITC-HA-BSA (20 µM). (**b**,**e**) PTX encapsulated within FITC-HA-BSA (PTX: BSA at a molar ratio of 2:1). (**c**,**f**) PTX encapsulated within FITC-HA-BSA + excess free HA (100-fold higher concentration of free HA compared to the conjugated HA). LysoTracker red DND99 (100 nM) was added 1 h before cell imaging. Hoechst 33342 (2 µg/mL) was added immediately before fluorescence microscopy analysis. Then, micrographs were taken at a X630 magnification using a Zeiss inverted Cell-Observer microscope.

The targeting selectivity of the NPs was then explored using two human ovarian cancer cells (Figure 7): SKOV3 that overexpressed CD44, and A2780 cells in which CD44 was undetectable [36].

FITC-HA-BSA NPs selectively targeted SKOV3 cells (CD44+), and were taken up via endocytosis, as evident from the yellow fluorescence (Figure 7a,b) indicating co-localization of LysoTracker red (red fluorescence) and the FITC-labeled NPs (green fluorescence). In contrast, no internalization of FITC-HA-BSA conjugates was observed in A2780 cells, which are devoid of CD44 (Figure 7d–f). Addition of a 100-fold excess of free HA to SKOV3 cells (Figure 7c) significantly reduced the yellow fluorescence signal of the NPs (compared to Figure 7b). Therefore, FITC-HA-BSA NPs were selectively targeted to the CD44 receptor of ovarian SKOV3 cancer cells (CD44+) but not to CD44-deficient A2780 cells.

#### 3.2.6. PTX Cytotoxicity

**Figure 8:**
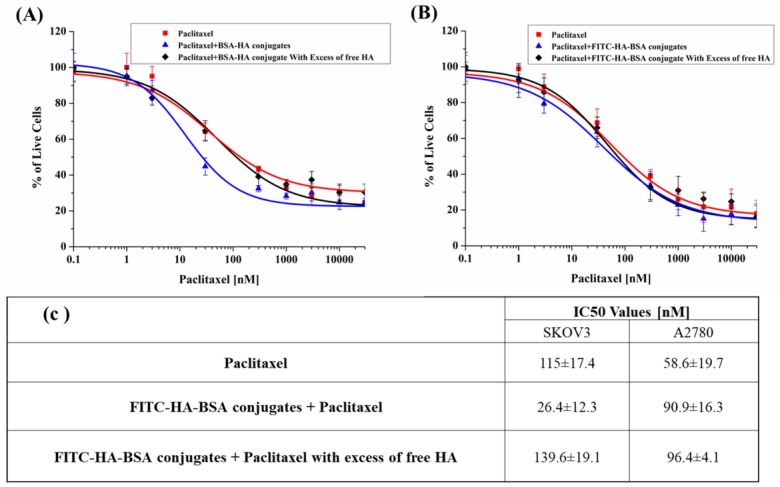
Survival of (**A**) SKOV3 and A2780 (**B**) cells incubated with PTX (■) vs. PTX encapsulated within FITC-HA-BSA conjugate NPs (▲) or with excess free HA (100-fold higher concentration of free HA compared to the conjugated HA) (◊). (**C**) Summary of the IC_50_ values.

The cytotoxic activity and selectivity of these PTX-loaded NPs was tested in SKOV3 ovarian carcinoma cells compared to A2780 cells. The cytotoxic activity was evaluated by determining cell viability after incubation with drug-loaded NPs using standard colorimetric XTT assays. Figure 8 depicts the fraction of live cells as a function of PTX concentration. Three samples were tested: free PTX, PTX-loaded within FITC-HA-BSA conjugate NPs, and PTX-loaded within FITC-HA-BSA conjugates with excess of free HA competing for CD44 receptor binding.

Figure 8a shows that PTX-loaded FITC-HA-BSA NPs were significantly more cytotoxic than free PTX towards SKOV3 cells (IC_50_: 26.4 ± 12.3 nM, and 115.0 ± 17.4 respectively, *p*-value = 0.05). The cytotoxicity of encapsulated PTX in SKOV3 cells was considerably decreased following the addition of free HA (IC_50_: 139.6 ± 19.1 nM, *p*-value = 0.04), bringing it back to about the IC_50_ value of free PTX, thereby providing additional indication for the CD44-mediated internalization of the FITC-HA-BSA NPs. In contrast, Figure 8b shows that in the absence of the CD44 receptor (A2780 cells), the cytotoxicity of encapsulated PTX and free PTX was low and nearly identical, with no significant difference (IC_50_: 90.9 ± 16.3 and 58.6 ± 19.7 nM, respectively, *p-*value = 0.36). Moreover, addition of excess free HA to A2780 ovarian cancer cells did not affect the cytotoxicity of encapsulated PTX.

## 4. Discussion

FITC-HA-BSA NP is a vehicle for simultaneous, targeted cancer therapeutics and diagnostics. By combining HA with SA, a self-assembling biodegradable block-copolymer, made of natural body components was created, in which HA also acts as the homing ligand for cancer cells exhibiting CD44 overexpression. The conjugation of FITC (and in the future, other diagnostic tracers suitable for in vivo imaging) to the NP formed a diagnostic imaging platform. The FITC label was covalently linked to HA to assure that the imaging tag is a direct indication of the selective binding of the NP to a cancer cell via the targeting component, HA. Entrapment of a chemotherapeutic drug within the self-assembled NPs created a model targeted theranostic delivery system. SA is an abundant globular blood protein; we recently introduced the potential of HA-SA Maillard conjugates for targeting CD44-overexpressing cancers [36]. In the present study, we further assessed the potential of such NPs towards multifunctional theranostics. By conjugating HA to BSA, we formed a self-assembling, actively targeted and biocompatible polymeric nanocarrier, which can entrap well-established hydrophobic cytotoxic drugs such as PTX [36]. Conjugation via the Maillard reaction requires only mild heating without adding any toxic/carcinogenic chemical reagents (typically used in various NP synthesis protocols). The Maillard reaction was performed under controlled mild conditions to prevent the formation of polymeric brown pigments. Similarly, a previous publication from our lab showed that Maillard reaction conjugates of casein and maltodextrin formed nanocapsules capable of entrapping hydrophobic nutraceuticals like vitamin D [24].

Compared to free hydrophobic drugs, drug-loaded NPs generally have several advantages such as longer circulation time, higher tissue penetration and high surface to volume ratio for targeting purposes [71,72]. Determining the size of our NP (by DLS and Cryo-TEM) revealed that FITC-HA-BSA conjugates were mostly around 5–10 nm, presumably containing one molecule of BSA and a few HA molecules. FITC-HA-BSA self-organized, showing a CMC value of 1.1 µM. The larger aggregates observed (~10–20 nm) seemed to indicate self-assembled NPs containing tens of molecules of FITC-HA-BSA conjugates.

PTX is a lipophilic drug with a log P value of 3.96 (aqueous solubility of <10 µg/mL [73]). Therefore, solubilization of PTX is urgently required. Encapsulation of PTX occurs mostly via hydrophobic and van der Waals interactions with the hydrophobic domains of BSA. The association constant of PTX-BSA was found to be (8.6 ± 0.8) × 10^3^ M^−1^. Non-encapsulated PTX exhibited a mean diameter of ≥1000 nm by DLS, and formed crystalline aggregates of ≥50 µm, as seen by light microscopy, whereas, in the presence of FITC-HA-BSA conjugates, the NPs were mostly between 10 and 20 nm. FITC-HA-BSA conjugates effectively suppressed crystal growth up to 4:1 PTX:NPs molar ratio, which was thus determined to be the maximal LC. On a mass basis, this translates to 33 mg of PTX per gm of BSA, and was achieved with a remarkable EE of 96%. Using β-casein to encapsulate PTX for oral delivery, we previously found an association coefficient of 9.6 × 10^3^ M^−1^, LC of 40 mg PTX per gm of protein, and EE of ~95% [74]. We have recently shown that the protein structure determines its efficacy in prevention of ligand crystal growth [74]. Zeta potential of non-encapsulated PTX was lower than FITC-HA-BSA NPs, indicating that PTX is unstable in aqueous media and that the encapsulation significantly improves its stability. The PTX release profile obtained is in accordance with previous findings in the literature [75,76,77]. It was observed here to be divided into two main phases: Initially, a burst release of only about 20% of the drug (most likely some incompletely entrapped drug) within 10 h, followed by a very slow drug release up to about 48 h. After 24 h, about 70% of the drug is still encapsulated, and even after 80 h, 40% of the drug remained entrapped within FITC-HA-BSA NPs. These are encouraging results, as EPR of long circulating NPs is taking place within approximately 24 h [70]. Hence, based on these results, about 70% of the drug should reach the tumor and not be prematurely released in the blood. Once in the tumor cells, the HA-BSA NPs will be proteolytically degraded inside the endo-lysosomes, thereby releasing the drug and eradicating the tumor cell. The stability of FITC-HA-BSA NPs was measured as the relative scattering intensity following mixing with potentially disruptive solutions: The stability of FITC-HA-BSA NPs encapsulating PTX was studied in the presence of 50% FBS and in the presence of SDS, which is known to induce micelle disruption. The light scattering intensity of the sample was around 100% of its initial value (immediately after mixing with the disruptive solutions) for 3 h in the presence of FBS and SDS.

Selective targeted delivery of theranostic NPs to the tumor site is critical for both diagnostic localization and therapy [2,3,72,78]. Receptor-mediated endocytosis is possible within the size range of 10–60 nm, whereas NPs smaller than 200 nm are suitable for the EPR effect [2,4,12,79]. A hydrophilic exterior and small size (<200 nm) diminish NPs uptake by the reticuloendothelial system (RES) [80]. Hence, in terms of size and structure, the PTX-loaded FITC-HA-BSA NPs developed herein should be suitable for passive targeting followed by active endocytosis via binding to CD44. Previous studies have utilized HA for targeting CD44-overexpressing cancer cells [34,81]. Several approaches have been reported to form such targeted nanovehicles, including HA-drug conjugates [34,82,83,84], cross-linked HA particles [85], polymeric NPs decorated with HA [86,87], including layer-by-layer formed NPs [85], and HA-grafted lipid-based vehicles. Different delivery systems combining PTX and HA have been previously evaluated for targeting CD44-overexpressing tumor cells. Rivkin et al., encapsulated PTX with lipids that self-assemble into NPs coated with hyaluronan and reported a diameter of ~300 nm with 10-fold accumulation in the tumor compared to conventional PTX formulation [86]. In the current study, we evaluated the selective targeting of CD44−overexpressing ovarian cancer cells using FITC-HA-BSA NPs. SKOV3 cells (CD44+), and A2780 cells (CD44−) were incubated with fluorescent FITC-HA-BSA NPs; FITC-HA-BSA NPs were selectively internalized via a CD44−dependent, receptor-mediated endocytosis by SKOV3 cells, but not by cognate A2780 cells, lacking CD44. Co-localization of the red (LysoTracker) and green (FITC-HA-BSA) fluorophores, appearing as yellow fluorescence evidenced the endocytosis of FITC-HA-BSA NPs within endosomes. Moreover, addition of competing free HA markedly decreased the yellow fluorescence signal, demonstrating that the internalization of these NPs occurred via the CD44 receptor. As mentioned in Section 2.2.14, the 100-fold higher HA concentration was safe for the cells. Furthermore, not only were the cells unharmed in the presence of excess HA, but they were also highly protected against the potent cytotoxic activity of PTX (see Figure 8). A similar pattern was observed in the NPs containing PTX; PTX did not interfere with the CD44-dependent internalization of the PTX-loaded conjugate NPs.

The binding affinity of HA to the CD44 receptor is dependent on the molar mass of HA; the binding to the CD44 receptor and uptake into SKOV3 cells was previously shown to increase with the size of HA above 10 monosaccharide units [87]. The minimal length of the HA oligomer for internalization was 4–8 monosaccharides [87]. In the current study, we employed an HA of ~6.4 kDa with approximately 34 monosaccharide units. This molecular mass was selected for several considerations: (i) low MW (<2.5 kDa) HA oligomers have been reported to enhance angiogenesis [88]; (ii) binding avidity to CD44 increases with HA chain length, and it was previously reported that oligomers of ~20–38 monosaccharides were simultaneously bound to two CD44 receptors and the avidity was ~3-fold higher than that of smaller oligomers [89]. Binding to several CD44 receptors by different HA chains attached to the same NP creates a sufficiently strong avidity for effective targeting and internalization [90]; (iii) selecting HA oligosaccharides which are sufficiently long to bind to more than a single CD44 receptor but too short to be bound by the HARE receptor in the liver (preferably <10 kDa), may facilitate the construction of an HA-based CD44-targeted nanocarrier that evades hepatic elimination but accomplishes passive targeting of the tumor, and selective active uptake into the tumor cells [87].

Finally, encapsulated PTX was significantly more cytotoxic than unencapsulated PTX, towards SKOV3 cells (IC_50_: 26.4 ± 12.3 nM and 115.0 ± 17.4, respectively, *p*-value = 0.05). The selective cytotoxic effect of encapsulated PTX will enable, in the future, to decrease the required chemotherapeutic dose of PTX, hence would markedly decrease its side effects. Here too, the addition of competing free HA to SKOV3 cells resulted in much lower cytotoxicity of encapsulated PTX, similar to that of free PTX (IC_50_: 139.6 ± 19.1 nM, compared to 115.0 ± 17.4; *p*-value = 0.04). This competitive suppression by free HA establishes the CD44-dependent selective cytotoxicity of the encapsulated PTX mediated by HA. In A2780 cells (CD44−), the IC_50_ values of encapsulated and unencapsulated PTX were similar (90.9 ± 16.3 and 58.6 ± 19.7 nM, respectively, *p*-value = 0.36). Addition of excess free HA to A2780 cells did not affect the cytotoxic activity of encapsulated PTX. These findings may have important clinical and therapeutic implications, as PTX toxicity often requires lowering the dose or discontinuing therapy, in spite of some positive clinical responses [91]. This cytotoxic treatment with PTX-loaded NPs that is specifically targeted to the tumor should reduce the untoward side-effects such as myelosuppression, peripheral neurotoxicity, mucositis and alopecia that are associated with i.v. administration of free PTX in the treatment of various malignancies. The natural next stage of the current research will be to explore the biodistribution and efficacy of the FITC-HA-BSA NPs theranostic delivery system in both diagnostic localization and tumor eradication *in vivo*.

## 5. Conclusions

HA was first covalently attached to a model diagnostic tracer, FITC, and then to BSA via the Maillard reaction. The size of the FITC-BSA-HA conjugates was 5–10 nm, and their self-assembled NPs were 10–20 nm. Based on DLS analysis, light microscopy and TEM results, PTX alone formed micrometer size crystalline aggregates, while encapsulation of PTX within FITC-HA-BSA conjugates formed NPs of 10–60 nm. Receptor-mediated endocytosis of FITC-HA-BSA conjugates to human ovarian cancer SKOV3 cells was via the CD44 receptor. The IC_50_ value of PTX-loaded FITC-HA-BSA conjugates was significantly lower in SKOV3 cells overexpressing CD44 than in A2780 cells devoid of CD44. Competition with excess free HA eliminated this IC_50_ value difference, hence further corroborating the selective CD44-mediated internalization of FITC-HA-BSA conjugates. The fact that the NPs enter the cell via endocytosis should enable them to evade MDR efflux transporters in MDR cancer cells, hence warranting their evaluation in a dedicated study aimed at overcoming MDR. Collectively, our current experimental results show that these biocompatible and biodegradable theranostic NPs, constructed of natural body components, may serve as a new platform for selective delivery of encapsulated hydrophobic chemotherapeutics. In addition, this nano delivery system may also encapsulate combinations of drugs and MDR modulators to reverse specific chemoresistance modalities and effectively overcome drug-resistant cancers. Following substitution of FITC with a tracer for in vivo diagnostics, such theranostic or even quadrugnostic nanovehicle platforms may be further studied in vivo for selective targeting of tumors and their distant metastases overexpressing CD44, for diagnostic localization purposes, and for targeted therapy of cancers, e.g., ovarian cancer and other malignancies, including those that frequently display chemoresistance.
